# Inhibition of Corneal Neovascularization with the Combination of Bevacizumab and Plasmid Pigment Epithelium-Derived Factor-Synthetic Amphiphile INTeraction-18 (p-PEDF-SAINT-18) Vector in a Rat Corneal Experimental Angiogenesis Model

**DOI:** 10.3390/ijms14048291

**Published:** 2013-04-16

**Authors:** Chien-Neng Kuo, Chung-Yi Chen, San-Ni Chen, Lin-Cheng Yang, Li-Ju Lai, Chien-Hsiung Lai, Miao-Fen Chen, Chia-Hui Hung, Ching-Hsein Chen

**Affiliations:** 1 Department of Ophthalmology, Chang Gung Memorial Hospital, No.6, W. Sec., Jiapu Rd., Puzi City, Chiayi County 61363, Taiwan; E-Mails: k771376@adm.cgmh.org.tw (C.-N.K.); lynnlai@cgmh.org.tw (L.-J.L.); oph4557@adm.cgmh.org.tw (C.-H.L.); 2 Chang Gung University College of Medicine, No.259, Wenhua 1st Rd., Guishan Township, Taoyuan County 33302, Taiwan; E-Mails: miaofen@adm.cgmh.org.tw (M.-F.C.); q22016@yahoo.com.tw (C.-H.H.); 3 Chang Gung University of Science and Technology, No.2, W. Sec., Jiapu Rd., Puzi City, Chiayi County 61363, Taiwan; 4 Department of Ophthalmology, Changhua Christian Hospital, Yun Lin Branch, No.375, Shichang S. Rd., Xiluo Township, Yunlin County 64866, Taiwan; 5 School of Medical and Health Sciences, Fooyin University, No.151, Jinxue Rd., Daliao Dist., Kaohsiung City 83102, Taiwan; E-Mail: xx377@fy.edu.tw; 6 Department of Ophthalmology, Changhua Christian Hospital. No.135, Nanxiao St., Changhua City, Changhua County 50006, Taiwan; E-Mail: 108562@cch.org.tw; 7 School of Medicine, Chung-Shan Medical University, Taichung City 50000, Taiwan; 8 Gene Therapy Laboratory, E-DA Hospital, I-Shou University, No.1, Sec. 1, Syuecheng Rd., Dashu District, Kaohsiung City 84001, Taiwan; E-Mail: lcyang1@ms13.hinet.net; 9 Department of Radiation Oncology, Chang Gung Memorial Hospital, No.6, W. Sec., Jiapu Rd., Puzi City, Chiayi County 61363, Taiwan; 10 Department of Dermatology, Chang Gung Memorial Hospital, No.6, W. Sec., Jiapu Rd., Puzi City, Chiayi County 61363, Taiwan; 11 Department of Microbiology, Immunology and Biopharmaceuticals, College of Life Sciences, National Chiayi University, Chiayi City 60004, Taiwan

**Keywords:** bevacizumab, PEDF, VEGF, bFGF, cornea, angiogenesis, SAINT-18

## Abstract

Bevacizumab, a 149-kDa protein, is a recombinant humanized monoclonal antibody to VEGF. PEDF, a 50-kDa glycoprotein, has demonstrated anti-vasopermeability properties. In this study, we demonstrated that the combination of bevacizumab and plasmid pigment epithelium-derived factor-synthetic amphiphile INTeraction-18 (p-PEDF-SAINT-18) has a favorable antiangiogenic effect on corneal NV. Four groups (Group A: 0 μg + 0 μg, B: 0.1 μg + 0.1 μg, C: 1 μg + 1 μg, and D: 10 μg + 10 μg) of bevacizumab + p-PEDF-SAINT-18 were prepared and implanted into the rat subconjunctival substantia propria 1.5 mm from the limbus on the temporal side. Then, 1 μg of p-bFGF-SAINT-18 was prepared and implanted into the rat corneal stroma 1.5 mm from the limbus on the same side. The inhibition of NV was observed and quantified from days 1 to 60. Biomicroscopic examination, western blot analysis and immunohistochemistry were used to analyze the 18-kDa bFGF, 50-kDa PEDF and VEGF protein expression. No inhibition activity for normal limbal vessels was noted. Subconjunctival injection with the combination of bevacizumab and p-PEDF-SAINT-18 successfully inhibited corneal NV. The bFGF and PEDF genes were successfully expressed as shown by western blot analysis, and a mild immune response to HLA-DR was shown by immunohistochemistry. We concluded that the combination of bevacizumab and p-PEDF-SAINT-18 may have more potent and prolonged antiangiogenic effects, making it possible to reduce the frequency of subconjunctival bevacizumab administration combined with a relatively safe profile and low toxicity.

## 1. Introduction

NV or angiogenesis, the formation of new blood vessels, is a normal process that accompanies tissue growth, reproduction and the repair of damaged tissue during the process of wound healing. Corneal NV can occur physiologically or pathologically and usually leads to vision impairment [1,2]. Several natural and synthetic angiogenesis inhibitors have shown beneficial effects in experimental animal studies, such as topical corticosteroids, suleparoide (heparan sulfate) [3], thalidomide [4], suramin [5] and genistein [6], are effective in inhibiting corneal NV [7,8]. However, the pathogenesis of corneal NV has not yet been fully defined; further investigation is needed to determine whether the anti-angiogenic therapy is effective for the treatment of actively growing or established corneal angiogenesis.

VEGF is a critical mediator of NV, and the level of VEGF in the vitreous is highly correlated with the growth of new blood vessels in eyes with diabetic retinopathy and choroidal NV [9–11]. Anti-VEGF has been associated with the inhibition of iris and corneal NV and suppression of the formation of new retinal vessels in primates [12–14]. Several antiangiogenic compounds that target VEGF and VEGF receptors are currently being developed, and some are available as tumor therapies.

Bevacizumab (Avastin; Genentech, San Francisco, CA, USA) is a humanized monoclonal antibody to VEGF that is approved by the U.S. Food and Drug Administration for the treatment of metastatic colorectal cancer [15]. This drug is now used off-label to treat the ocular system through the inhibition of pathologic blood vessels and decreased vascular permeability [16,17]. Recently, several reports have shown that subconjunctival bevacizumab inhibits corneal NV in animal models [18] and that topically applied bevacizumab limits corneal NV after chemical burns [19]. However, the estimated half-life of subconjunctival bevacizumab may be short. According to one report, the vitreous half-life of 1.25 mg of intravitreal bevacizumab is 4.32 days in the rabbit eye [20]. Therefore, repeated ocular injections may be required, especially if upregulated angiogenesis continues.

PEDF, a 50-kDa glycoprotein initially isolated from the conditioned media of retinal pigment epithelial (RPE) cells, has demonstrated neurotrophic, neuronotrophic, neuroprotective, gliastatic, anti-tumorigenic and anti-vasopermeability properties [21,22]. A possible role for PEDF in the regulation of ocular NV was suggested as the molecule was detected in the vitreous and the aqueous humor and shown to be one of the most potent known anti-angiogenic proteins found in humans [21,23]. Notably, PEDF inhibits the VEGF-induced proliferation and migration of microvascular endothelial cells [24] and the bFGF-induced capillary morphogenesis of endothelial cells [25–28] through Fyn [29]. Matsui T. *et al.*[30] reported that a PEDF-derived peptide inhibits corneal angiogenesis by suppressing VEGF expression. In addition, PEDF is the most potent natural inhibitor of angiogenesis in the mammalian eye. The following is a model modified from those of Aplin *et al*. [25] and Conway *et al.*[27], which demonstrated that the binding of vascular endothelial growth factor (VEGF) with its receptors results in the self-phosphorylation (P) of the receptors, which in turn leads to a series of intracellular events that trigger angiogenesis. In this case, integrins are associated with protein tyrosine phosphatase (PTP). However, the activation of integrins in adhering cells may activate protein kinases, thereby triggering a series of downstream events to inhibit the angiogenesis induced by VEGF. The binding of PEDF with integrins, directly and/or indirectly via ECM, leads to conformation changes of the integrin or collagen-integrin complexes, causing phosphatases to dissociate and then reassociate with the VEGF receptors. This PEDF-integrin complex may also inactivate the protein kinases. The dephosphorylation of receptors or their downstream factors stops signal transduction, thereby blocking angiogenesis [28]. VEGF signaling influences the repair of corneal nerves, which was demonstrated by the presence of VEGF and VEGF receptors in the trigeminal ganglia and by the abrogation of VEGF signaling reducing nerve growth *in vitro* and *in vivo*[31]. Although a PEDF-derived peptide inhibits corneal angiogenesis by suppressing VEGF expression [30], PEDF, which has neurotrophic activity, has the additional advantage of preserving neurons from the damage often caused by vascular diseases in the nervous system [28].

We previously showed that SAINT-18 was capable of directly delivering genes to the ocular surface via subconjunctival injection and that it delivered sustained high levels of gene expression *in vivo* to inhibit angiogenesis. This effect may last longer than that of bevacizumab alone because the PEDF protein could be detected on day 60 after the transfection with p-PEDF-SAINT-18 [30]. In this study, we were interested in understanding the antiangiogenic effect of the combination of bevacizumab and p-PEDF-SAINT-18 on corneal NV.

## 2. Results and Discussion

### 2.1. Biomicroscopic Examinations of Corneal NV

**Figure 1:**
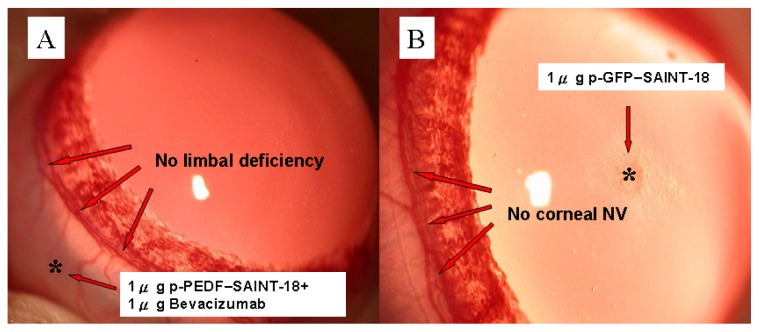
(**A**) Limbal deficiency test: 1 μg of bevacizumab + 1 μg of p-PEDF-SAINT-18. No inhibition of the normal limbal vessels was noted on day 15; (**B**) The effect of GFP on corneal neovascularization: The 1 μg of p-SAINT-18 gene complex was implanted into the corneal stromal pocket. No limbal vessels began sprouting into the cornea on day 15.

**Figure 2:**
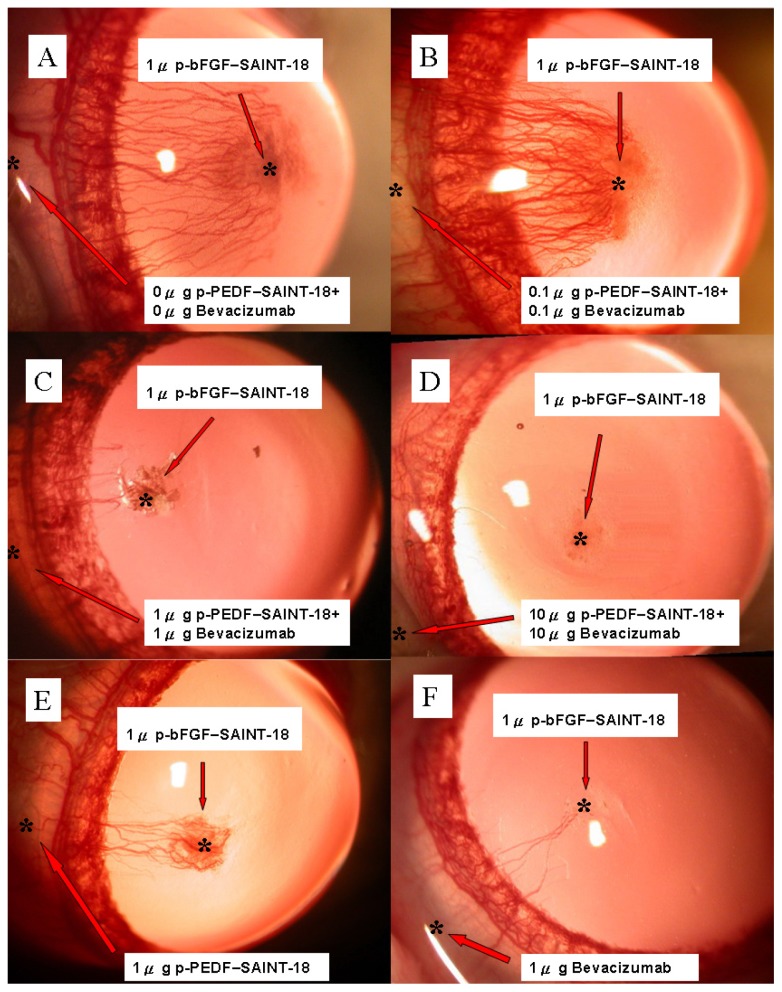
Slit-lamp photographs of Sprague-Dawley rat corneas showing corneal NV for the four experimental groups on day 15. (**A**) 0 μg + 0 μg of bevacizumab + p-PEDF-SAINT-18, respectively (control group; the substitute was purified water); (**B**) 0.1 μg + 0.1 μg of bevacizumab + p-PEDF-SAINT-18, respectively; (**C**) 1 μg + 1 μg of bevacizumab + p-PEDF-SAINT-18, respectively; (**D**) 10 μg + 10 μg of bevacizumab + p-PEDF-SAINT-18, respectively. * 1 μg of the p-Bfgf-SAINT-18 complex; (**E**) Subconjunctival administration of 1 μg of p-PEDF-SAINT-18 alone; (**F**) Subconjunctival administration of 1 μg of bevacizumab alone.

Gross examination of our rat corneal model seemed to show no interference with the wound healing process and no corneal limbal deficiency was induced by bevacizumab + p-PEDF-SAINT-18 (Figure 1A). Another control, 1 μg p-GFP-SAINT-18, was evaluated and compared (Figure 1B). The inhibition of NV in the treatments with 1 μg bevacizumab alone and 1 μg p-PEDF-SAINT-18 alone was shown on day 15 (Figure 1C,D). Forty-eight rats (48 eyes) were divided equally into three experimental groups (Group B: 0.1 μg + 0.1 μg, C: 1 μg + 1 μg, and D: 10 μg + 10 μg of bevacizumab + p-PEDF-SAINT-18, respectively) and one control group (Group A: 0 + 0 μg of bevacizumab + p-PEDF-SAINT-18, respectively, with purified water as the substitute). Biomicroscopic examinations revealed that the corneal epithelium healed within 24 h of the surgery. Corneal edema and limbal injection were noted in all corneas. The limbal vessels began sprouting into the cornea on postoperative day 3. Our previous study demonstrated that corneal NV was induced dose-dependently by 1 μg of the p-bFGF-SAINT-18 complex and that NV reached a maximum on days 12–18 in the control group, followed by progressive regression. [32] The NV response was intense, localized, and reproducible. The maximal growth of the NV is shown in Figure 2. Compared with the control group, there was significant inhibition of NV in groups C and D.

**Figure 3:**
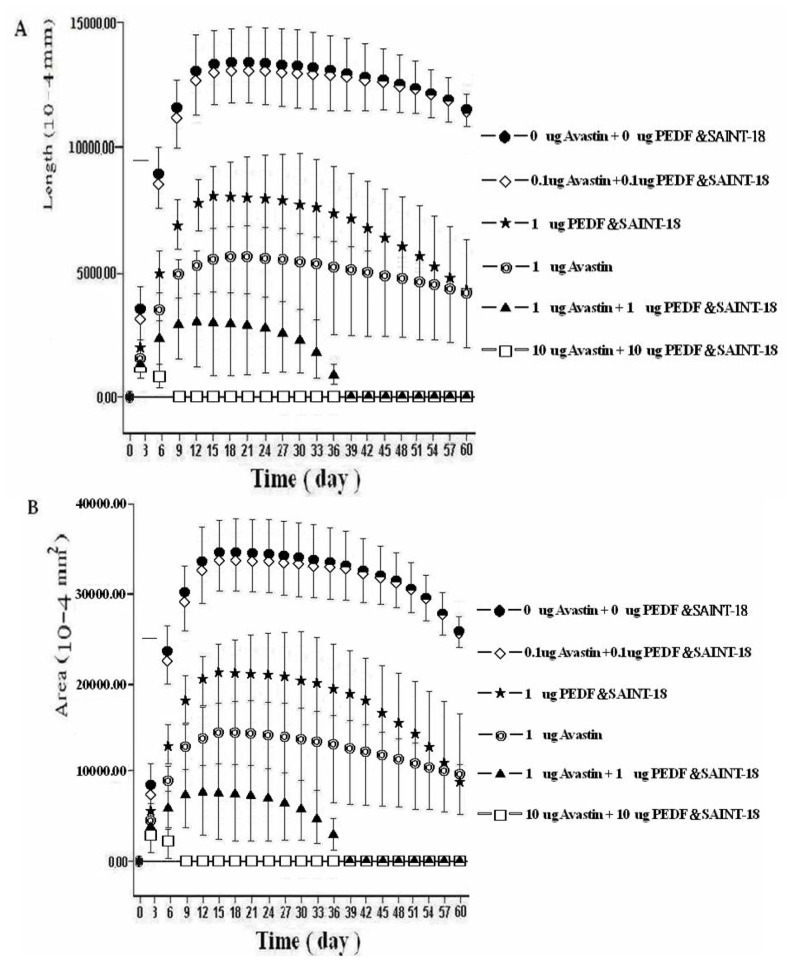
The mean lengths (**A**) and areas (**B**) of the corneal NV. The angiogenesis induced by 1 μg p-bFGF-SAINT-18 and in the treatments of the six different groups by subconjunctival injection. The vertical bars denote the standard deviation of the mean.

However, the standard deviation was high (e.g., group C, 5518 ± 2274 × 10^–4^ mm; group D, 1610 ± 792 × 10^–4^ mm) on day 6 after the transfection. Data from the four groups were compared using repeated measures ANOVA. The results of the length data were *F* = 1025.17 (*p* < 0.001 between the control and either group C or D, but *p* > 0.05 between the control and group B). The results of the area data were *F* = 172.04 (*p* < 0.001 between the control and either group C or D, but *p* > 0.05 between the control and group B) (Figure 3). The inhibition of NV in the treatment with 1 μg of bevacizumab alone was greater than that in the treatment with 1 μg of p-PEDF-SAINT-18 alone (*p* < 0.05). However, on day 60, p-PEDF-SAINT-18 showed a similar antiangiogenic effect to that of bevacizumab, suggesting that the antiangiogenic effect of p-PEDF-SAINT-18 may last longer than that of bevacizumab but be less potent during the early period of the treatment. In addition, we also evaluated the antiangiogenic effect among the three groups (1 μg of bevacizumab alone, 1 μg of p-PEDF-SAINT-18 alone and 1 μg of bevacizumab + 1 μg of p-PEDF-SAINT-18). The results of length data were *F* = 474.69 (*p* < 0.001 between 1 μg of bevacizumab alone and 1 μg of p-PEDF-SAINT-18 alone). Moreover, the results of the length data were *F* = 471.99 (*p* < 0.001) between 1 μg of p-PEDF-SAINT-18 alone and 1 μg of bevacizumab +1 μg of p-PEDF-SAINT-18 but *F* = 127.32 (*p* > 0.05) between 1 μg of bevacizumab alone and 1 μg of bevacizumab + 1 μg of p-PEDF-SAINT-18. The results of the area data were *F* = 205.08 (*p* < 0.001) between 1 μg of bevacizumab alone and 1 μg of p-PEDF-SAINT-18 alone. Importantly, the results of the area data were *F* = 105.55 (*p* < 0.001) between 1 μg of p-PEDF-SAINT-18 alone and 1 μg of bevacizumab +1 μg of p-PEDF-SAINT-18 and *F* = 71.98 (*p* < 0.05) between 1 μg of bevacizumab alone and 1 μg of bevacizumab +1 μg of p-PEDF-SAINT-18 (Figure 3).

### 2.2. Western Blot Analysis

**Figure 4:**
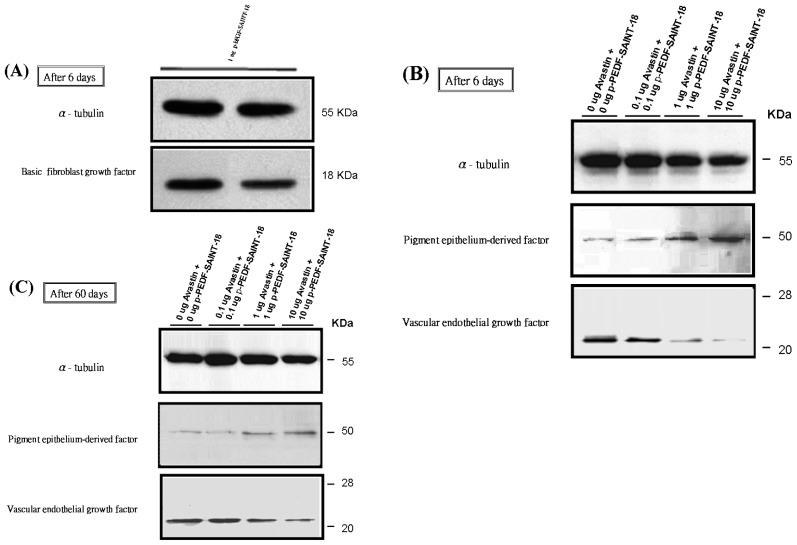
Levels of bFGF, VEGF and PEDF proteins estimated by western blot analysis. The α-tubulin band was used as the control for normalization. (**A**) The bFGF bands (molecular weight, approximately 18 kDa) produced with 1 μg of the p-bFGF-encoding plasmid after day 6; (**B**) Visualization of the PEDF bands (molecular weight, approximately 50 kDa) produced with 0 μg, 0.1 μg, 1 μg, and 10 μg of PEDF-encoding plasmid. This band was also noted in the 0 μg PEDF group due to endogenous PEDF. The inhibition of VEGF expression was more significant in the 10 μg + 10 μg bevacizumab + p-PEDF-SAINT-18 group; (**C**) On day 60 after administration, the PEDF and VEGF levels within the corneal and subconjunctival substantia propria were determined.

On day 6 after transfection, the rats in each group were killed with an overdose of pentothal, and the corneal samples were removed along the contour of the NV. The α-tubulin band was used as the control for normalization. The bFGF bands (molecular weight, approximately 18 kDa) were detected in the protein extracts from the groups treated with 1 μg p-bFGF using a rabbit anti-human bFGF polyclonal antibody (Figure 4A). The PEDF bands (molecular weight, approximately 50 kDa) produced with 0 μg, 0.1 μg, 1 μg and 10 μg of the PEDF-encoding plasmid was visualized. This band is also noted in the 0 μg PEDF group due to the presence of endogenous PEDF. The levels of bound VEGF protein were also estimated by western blot analysis on days 6 and 60 (Figure 4B,C).

### 2.3. Histology

**Figure 5:**
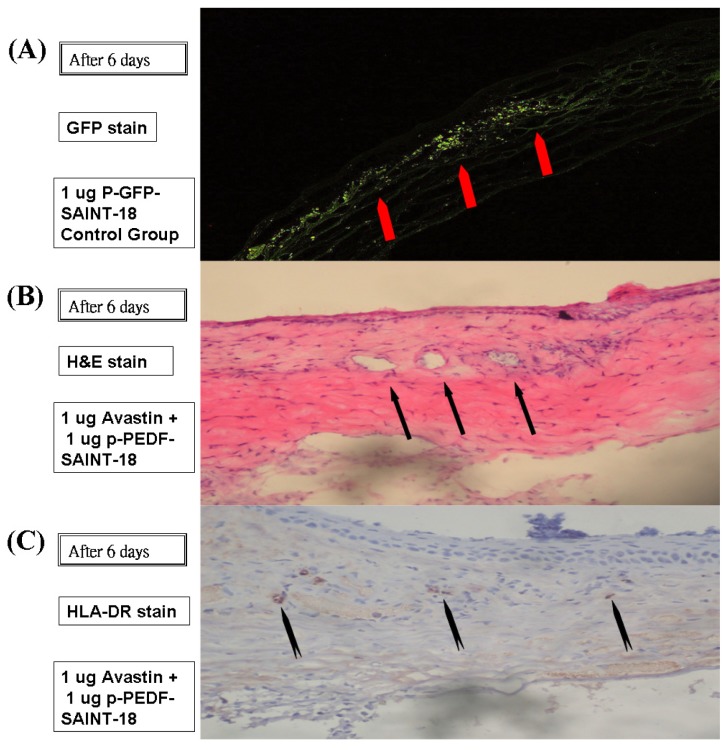
The tissue was histologically evaluated on day 6 after administration. (**A**) GFP was expressed within the corneal intrastromal layer and keratocytes after the implantation of p-GFP-SAINT-18. No vascular lumen was noted; (**B**) The intervening stroma displayed cells, edema, a mononuclear inflammatory response, and numerous vascular lumens (indicated by arrows) after hematoxylin and eosin staining in the corneal and subconjunctival substantia propria (100× magnification); (**C**) Inflammation was detected by the presence of macrophages and other inflammatory cells, such as lymphocytes, by immunohistochemical staining (anti-rat HLA-DR antibody) in the corneal and subconjunctival substantia propria (100× magnification).

Six days after administration, the successful gene expression of p-GFP-SAINT-18 within the corneal was histologically evaluated (Figure 5A). A vast number of capillaries appeared in the corneal stroma, running from the limbal blood vessels up to the p-bFGF-SAINT-18 implant (Figure 5B). Inflammation could be detected by the presence of macrophages and other inflammatory cells, such as lymphocytes, but a mild immune response caused by HLA-DR was shown by IHC staining (Figure 5C).

Ocular NV diseases are major contributors to blindness around the world. Angiogenesis in the eye may be the result of an imbalance between stimulatory and inhibitory factors that presumably results from the elevated expression of local angiogenic factors induced by ischemia. Several substances are used for inducing corneal NV, such as VEGF and bFGF [33]. VEGF and its receptors play a vital role in normal and pathologic angiogenesis [34]. It is an important signaling protein that promotes several steps of angiogenesis, including differentiation, proteolytic activity, proliferation and endothelial cell migration [35]. The bFGF that was utilized in this study has been used extensively in corneal angiogenesis models. It is widely expressed in developing and adult tissues during cellular differentiation, angiogenesis, mitogenesis and wound repair.

Recent studies have shown that the subconjunctival administration of bevacizumab effectively inhibits corneal NV in experimental models or clinical trials [18,19,36]. However, repeated administration or repeated injections with other modalities may be required for a better effect because bevacizumab has a short half-life. We have observed that the transgene, PEDF, can be stably expressed for more than three months [37]. Thus, we hypothesized that group D in this study would show the greatest regression of corneal NV because the combined treatment with bevacizumab and p-PEDF-SAINT-18 could block various proangiogenic growth factors more effectively through multiple mechanisms. First, bevacizumab can bind to and inhibit the biological activity of all five human VEGF-A isoforms: VEGF115, VEGF121, VEGF165, VEGF189, and VEGF206 [18]. Second, the PEDF-integrin complex may inactivate the protein kinase. The dephosphorylation of receptors or their downstream factors stops signal transduction, thereby blocking angiogenesis [25,27]. The results obtained through this study were similar to what we expected initially. Here, we observed less NV in group D than in the control group (group A). The H&E staining supported these results.

Compared with subconjunctival injection, other traditional methods, such as (1) intracameral and intravitreous injections, are more invasive to the eye ball, thus strong technical skills are needed by a physician using intracameral and intravitreous injections. They may cause penetrating wounds into the anterior chamber of the eye ball, and the risks of ocular infection and endophthalmitis are much higher. Cataract formation is also another severe complication of intraocular procedures. The large volume of non-viral particles may cause aqueous obstruction within the trabecular meshwork; (2) Topical application includes the difficulties of formulation, low water solubility and low stability in solution with a consequent susceptibility to the loss of bioactivity during long-term storage [38]. Additionally, long-term topical administration of bevacizumab may induce epitheliopathies and descemetocele [39,40]. Furthermore, the risk of systemic side effects may be higher with topical administration than with injection into the subconjunctivae. Because the conjunctival blood vessels do not form a tight junction barrier [41], subconjunctival injection is also an effective mode of administration for intraocular neovascular diseases. In general, a drug injected into the subconjunctival space has two fates: direct transscleral delivery into intraocular tissues or clearance via conjunctival blood and lymphatic flow [42–45]. Based on the reasons above, subconjunctival injection is considered rather than other traditional applications. However, subconjunctival injection could result in minor complications, such as subconjunctival hemorrhage, thinning or erosive changes to the conjunctiva and/or sclera.

During the early period of the subconjunctival administration, bevacizumab showed more significant antiangiogenic effects than p-PEDF-SAINT-18. These effects of bevacizumab were similar to the results reported in previous studies of different animal models [18,45,46]. On day 60, p-PEDF-SAINT-18 showed a similar antiangiogenic effect to that of bevacizumab, suggesting that the antiangiogenic effect of p-PEDF-SAINT-18 may last longer than that of bevacizumab but may be less potent during the early period of the treatment. One of the limitations of this study was its short duration; nonetheless, we observed that the PEDF transgene was stably expressed for more than three months, with the potential for longer periods of time, which should be studied. The expression of the gene is usually confined to the vicinity of the injection site. Concern about the possible side effects of the therapeutic gene over the long term could be quelled upon termination of the gene expression after disease recovery; the long-term effects require further evaluation.

## 3. Experimental Section

### 3.1. Animals

The animal experimental study was divided to two parts: In the first part, a total of 12 male Sprague-Dawley rats (300–350 g; NSC Animal Center, Taiwan) were used for the study of corneal limbal deficiency (The results were expressed in Figure 1A) and a total of 12 male Sprague-Dawley rats were used for the study of GFP expression (The results were expressed in Figure 1B). In the second part, a total of 72 male Sprague-Dawley rats were used in this study. Using the 5 experimental treatments (and 1 control group), every group contained 12 rats: (a) 0 μg of bevacizumab + 0 μg of p-PEDF-SAINT-18 including corneal p-bFGF-SAINT-18; (b) 0.1 μg of bevacizumab + 0.1 μg of p-PEDF-SAINT-18 including corneal p-bFGF-SAINT-18; (c) 1 μg of bevacizumab + 1 μg of p-PEDF-SAINT-18 including corneal p-bFGF-SAINT-18; (d) 10 μg of bevacizumab and 10 μg of p-PEDF-SAINT-18 group including corneal p-bFGF-SAINT-18; (e) 1 μg of p-PEDF-SAINT-18 including corneal p-bFGF-SAINT-18; and (f) 1 μg of bevacizumab including corneal p-bFGF-SAINT-18 (The results were expressed in Figure 2 and the corresponding group in Figure 3). All protocols and the treatment of the animals were in accordance with the Association for Research in Vision and Ophthalmology Statement for the Use of Animals in Ophthalmic and Vision Research. The animal protocol in this study was reviewed and approved by the Institutional Animal Care and Use Committee (IACUC), and the committee recognized that the proposed animal experiment followed the Animal Protection Law by the council of Agriculture, Executive Yuan, R.O.C. and the guidelines in the Guide for the Care and Use of Laboratory Animals as promulgated by the Institute of Laboratory Animal Resources, National Research Council, USA.

### 3.2. Naked DNA Vector

The bFGF and PEDF expression vectors pCMV-GFP, pCMV-bFGF and pCMV-PEDF were kindly provided by Dr M. H. Tai (Department of Medical Research, Kaohsiung Veteran General Hospital, Taiwan). The plasmid was purified commercially by Clone-E Therapeutics Inc. (Kaohsiung, Taiwan) and was endotoxin free. DNA was produced according to the proprietary process established by Clone-E Therapeutics Inc. The *E. coli* DH5α cells were purchased from Bioresource Collection & Research Center (Hsinchu, Taiwan). These cells carrying the plasmid were grown in an ampicillin-containing medium in a 5-L fermentor. The fermentation broth was subjected to a series of purification steps, including complete cell lysis, anion exchange, and gel filtration chromatography. The purified plasmid was dialyzed against a formulation buffer (The DNA plasmid formulation buffer is 10 mM phosphate containing 150 mM NaCl, pH 7.0). The DNA was quantified using UV absorbance. Agarose gel analysis showed primarily supercoiled plasmids with a small amount of nicked plasmid.

### 3.3. p-DNA-SAINT-18 Complex Preparation

The SAINT-18 (1-methyl-4-[*cis*-9-dioleyl]methylpyridinium-chloride) delivery system (Synvolux Therapeutics B.V. Groningen, Netherlands) was based on a cationic pyridinium head group, showing excellent bio-compatibility. The molecular structure of SAINT-18 was purchased from Synvolux Therapeutics B.V. Each vial was “ready to use” and contained 2 mL of SAINT-18 in water at a concentration of 0.75 mM. Before use, the SAINT-18 was vortexed thoroughly to minimize micelle-size, thereby increasing its complexing efficacy. The relative amounts of DNA to carrier for SAINT-18/DNA (1 μL of SAINT-18 per 1 μg of DNA phosphate) were allowed to form at room temperature. To minimize the loss experienced with liquid reagents during transfection into corneal tissue, the complex was partially dried by SpeedVac (SpeedVac Model SC110 + VLP120 Oil Vacuum Pump, Savant Instruments, Inc., Farmingdale, NY, USA) at ambient temperature for 60–90 min after the plasmid bFGF (1 μg) and PEDF [0 μg (with purified water as the substitute), 0.1 μg, 1 μg or 10 μg] had been complexed with SAINT-18. The partially dried form of the complex was drawn out of the Eppendorf tube by curettage and prepared for immediate implantation into the corneal pocket.

### 3.4. Corneal Pocket Assay

All surgical procedures were performed using sterile techniques. The corneal pocket implantation performed in this study was a modification of a previously described technique [37]. The rats were placed under general anesthesia with 3% isoflurane in an O_2_/room air mixture (1:1). As additional topical anesthesia, 0.4% benoxinate hydrochloride (Novesin, Ciba Vision, Hettlingen, Switzerland) was applied to the corneal surface. The eyes were proptosed by grasping the temporal limboconjunctival epithelium with a jeweler’s forceps, and a 30°–45° fan-shaped central-peripheral corneal intrastromal lamellar pocket (middle stroma depth; the distance of the inlet was 0.7–1.0 mm; the distance of the radius was 1.5–2.0 mm) was dissected with a surgical blade (Paragon No. 11, Maersk Medical, and Sheffield, UK) and an ophthalmic slit knife (Alcon Inc., Fort Worth, TX, USA). The pocket was extended 1.5 mm from the limbus. After the p-bFGF-SAINT-18 gene complex (1 μg) was implanted into the corneal stromal pocket in each eye using forceps and a blade, 0 + 0 μg, 0.1 + 0.1 μg, 1 + 1 μg and 10 + 10 μg of bevacizumab and p-PEDF-SAINT-18, respectively, were immediately delivered to the subconjunctival tissue 1.5 mm from the limbus via the indwelling cannula using a Hamilton syringe. The topical antibiotic ointment (0.3% gentamycin; Alcon Cusi, Camil Fabra, Masnou, Barcelona, Spain) was applied to the corneal surface to reduce irritation and prevent infection.

### 3.5. Visualization and Quantification of Corneal NV

Examinations were performed with a dissecting microscope, and the results were photographed. While the rats were under anesthesia, the eyes were proptosed, and the maximum vessel length and width in the NV region were measured with calipers. Photographs obtained during corneal angiogenesis assay were taken at a resolution of 640 × 480 pixels using a digital CoolPix 995 camera (Nikon, Chiyoda-ku, Tokyo, Japan). The operator was masked to the treatment group of each cornea. The areas containing blood vessels were traced on the computer monitor (FT Data Systems, Stanton, CA, USA). The area within the trace was calculated with image analysis software (Enhance 3.0; MicroFrontier, Des Moines, IO, USA) and is reported in square millimeters with the determinations confirmed at ×40 magnification. Three independent observers conducted the masked assessments.

### 3.6. Analysis of bFGF, VEGF and PEDF Protein Expression by Western Blot after Transfection

On days 6 and 60 after transfection, the rats in each group were killed with an overdose of thiopental sodium, and the fresh corneas were removed along the contour of NV using scissors. These specimens were homogenized by sonication in ice-cold lysis buffer (50 mM Tris, (pH 7.5), 150 mM NaCl, 2% Triton X-100, 100 μg/mL phenylmethylsulfonyl fluoride, 1 μg/mL aprotinin) and then centrifuged at 50,000× *g* for 30 min at 4 °C. The protein content of the supernatant was determined using the Bio-Rad Protein Assay system. An equal volume of sample buffer (2% sodium dodecyl sulfate (SDS), 10% glycerol, 0.1% bromophenol blue, 2% 2-mercaptoethanol, 50 mM Tris-HCl (pH 7.2)) was added to the sample. The proteins were separated by electrophoresis (NuPAGE Electrophoresis; Invitrogen, San Diego, CA, USA) on 15% SDS-polyacrylamide gels at 120 V for 90 min. They were then transferred to polyvinylidene difluoride membrane (0.45 μM pore size; Immobilon-P, Millipore, Billerica, MA, USA) in transfer buffer (50 mM Tris-HCl, 380 mM glycine, 1% SDS, 20% methanol) at 50 V for 60 min. The membrane was blocked with 5% nonfat dry milk in Tween-20 and Tris-buffered saline (TTBS; 0.1% Tween-20, 20 mM Tris-HCl, 137 mM NaCl, pH 7.4) for 60 min at room temperature. The membrane was then incubated with anti-bFGF (purified anti-human FGF-basic antibody; BioLegend, San Diego, CA, USA), anti-PEDF antibody (anti-human serpin F1/PEDF antibody; R&D Systems, Inc., Minneapolis, MN, USA) and anti-VEGF (Santa Cruz Biotechnology Inc. Dallas, TX, USA) for 90 min at room temperature. Each blot was washed three times for 10 min in TTBS, blocked with 5% nonfat dry milk in TTBS, and then incubated with horseradish-peroxidase-conjugated secondary antibody (1:1000; Transduction Laboratories) for 1 h at room temperature. The antibody labeling was detected by chemiluminescence (ECL, Amersham, GE Healthcare Biosciences P.O. Box 643065, Pittsburgh, PA, USA). Colored molecular-weight standards were run in parallel on each gel. The housekeeping gene α-tubulin was used as the control.

### 3.7. Histological Examination

On days 6 and 60 after transfection in group D, the rats were killed with an overdose of thiopental sodium; their eyes were then enucleated, fixed in paraformaldehyde, and frozen at −70 °C in OCT compound. The corneas were excised and cryostatically cut into 3–8-μm sections for hematoxylin and eosin (H&E) staining and immunohistochemistry (IHC). The excised corneal tissues for IHC were fixed in Bouin’s solution and embedded in paraffin. After the different sections had been cut (3 μm) and mounted on slides coated with poly-l-lysine, they were immunostained (ImmunoBlot, Invitrogen Corporation, Camarillo, CA, USA) with anti-rat HLA-DR antibody (Santa Cruz Biotechnology Inc., Santa Cruz, CA, USA).

### 3.8. Statistical Analysis

Repeated measures ANOVA (SPSS version 10.0 for Windows; SPSS Inc., Chicago, IL, USA) and the Bonferroni post hoc test were used to analyze the differences in the length and area of the corneal NV between pairs of groups. The length and area were measured every three days from day 0 to day 60. The values for each measurement were recorded as separate variables and defined as within-subject factors. The assigned groups were defined as between-subject factors. A value of *p* ≤ 0.05 was considered statistically significant.

## 4. Conclusions

In conclusion, we found that the combination of bevacizumab and p-PEDF-SAINT-18 may have more potent and prolonged antiangiogenic effects, making it possible to reduce the frequency of subconjunctival bevacizumab administration with a relatively safe profile and low toxicity.

## Abbreviations

PEDF: pigment epithelium derived factor
BFGF: basic fibroblast growth factor
SAINT-18: synthetic amphiphile interaction-18, 1-methyl-4-(*cis*-9-dioleyl) methylpyridinium-chloride
VEGF: vascular endothelial growth factor
GFP: green fluorescein protein
NV: neovascularization
